# Evaluation of Tooth Shade Selection With Conventional Methods and Artificial Intelligence-Integrated Methods: A Comparative Study

**DOI:** 10.7759/cureus.116086

**Published:** 2026-09-11

**Authors:** Rahul Das, Sheenam Markan, Mukesh Kumar, Megha Gupta, Sameer Chauhan, Prem S Parepalli

**Affiliations:** 1 Department of Dentistry, PKG Medical College and Hospital, Kolkata, IND; 2 Department of Conservative Dentistry and Endodontics, Government Dental College, Amritsar, IND; 3 Department of Oral and Maxillofacial Surgery, Shree Bankey Bihari Dental College, Ghaziabad, IND; 4 Department of Oral and Maxillofacial Surgery, Maharishi Markandeshwar College of Dental Sciences and Research, Maharishi Markandeshwar (Deemed to be University), Mullana, IND; 5 Department of Prosthodontics, K. M. Shah Dental College and Hospital, Sumandeep Vidyapeeth (Deemed to be University), Vadodara, IND; 6 Department of Conservative Dentistry and Endodontics, Sree Sai Dental College and Research Institute, Srikakulam, IND

**Keywords:** and dental, artificial intelligence, colorimetry, esthetics, spectrophotometry, tooth color

## Abstract

Introduction

Accurate tooth shade selection is fundamental for achieving optimal esthetic outcomes in restorative and prosthodontic dentistry. Conventional visual shade selection is widely used, but is susceptible to subjective variability arising from examiner experience, lighting conditions, and color perception. Artificial intelligence (AI)-integrated shade selection has emerged as a promising alternative, offering objective and reproducible color assessments. This study compared the diagnostic performance of conventional visual and AI-integrated tooth shade selection methods, using spectrophotometric evaluation as the reference standard.

Materials and methods

This prospective, single-center study included 55 participants who required tooth-shade assessment. Each participant underwent conventional visual shade selection using the VITA Classical A1-D4 and VITA Toothguide 3D-MASTER shade guides (VITA Zahnfabrik H. Rauter GmbH & Co. KG, Bad Säckingen, Germany), AI-assisted shade selection from standardized clinical photographs, and spectrophotometric evaluation using VITA Easyshade V (VITA Zahnfabrik H. Rauter GmbH & Co. KG, Bad Säckingen, Germany) as the reference standard. Agreement was assessed using Cohen's and weighted kappa statistics, color differences using CIEDE2000 (ΔE00), reliability using the intraclass correlation coefficient (ICC), and diagnostic performance using sensitivity, specificity, receiver operating characteristic (ROC) analysis, and area under the curve (AUC).

Results

The AI-integrated method demonstrated substantially higher agreement with the spectrophotometric reference than the conventional visual method (Cohen's κ: 0.75 vs. 0.43), with weighted κ values of 0.99 and 0.97, respectively. The exact shade agreement was 78.2% for the AI and 49.1% for the conventional visual assessment. The AI method exhibited excellent test-retest reliability (ICC 0.97), whereas intra- and inter-examiner ICCs for the conventional method were 0.71 and 0.74, respectively. The mean ΔE00 values were significantly lower for AI (1.42 ± 1.25) than for conventional visual assessment (1.76 ± 1.44), with a significant mean difference of −0.34 (95% CI: −0.57, −0.11; p = 0.020). AI achieved a higher diagnostic accuracy than the conventional method for detecting clinically perceptible shade mismatch (accuracy: 87.3% vs. 74.5%), with a sensitivity of 94.3% and specificity of 75.0%. ROC analysis demonstrated a superior discriminative ability for AI (AUC 0.932) compared to conventional visual assessment (AUC 0.895).

Conclusions

Artificial intelligence-integrated tooth shade selection demonstrated superior agreement, color accuracy, reliability, and diagnostic performance compared to conventional visual assessment when validated against spectrophotometric measurements. AI-assisted shade selection may serve as a reliable adjunct for objective color matching, improving esthetic predictability and consistency in restorative and prosthodontic practices. Larger multicenter studies are warranted to validate these findings and support broader clinical implementation.

## Introduction

Accurate tooth shade selection is a critical determinant of the esthetic success of restorative and prosthodontic treatment. The ability to reproduce the natural color of teeth directly influences the appearance, patient satisfaction, and long-term acceptance of dental restorations [1]. Despite advances in restorative materials, achieving an ideal color match remains challenging because tooth color is influenced by multiple factors, including translucency, surface texture, hydration, lighting conditions, surrounding oral tissues, and the visual perception of the observer [2]. Even minor discrepancies in shade selection can compromise esthetic outcomes, necessitating restoration remakes, increasing treatment costs, and reducing clinical efficiency. Consequently, accurate and reproducible shade selection has become an essential component of contemporary esthetic dentistry [1].

Traditionally, tooth shade selection has relied on visual comparison using standardized shade guides, such as the VITA Classical and VITA 3D-Master systems (VITA Zahnfabrik H. Rauter GmbH & Co. KG, Bad Säckingen, Germany). Although these methods are simple, inexpensive, and widely available, they are inherently subjective and are affected by examiner experience, color vision, fatigue, ambient lighting, and environmental conditions [3,4]. Recent advances in digital dentistry have significantly improved the objectivity and reproducibility of tooth-shade selection. Digital technologies, including intraoral scanners and spectrophotometric systems, have demonstrated higher reliability and reduced operator-dependent variability than conventional visual shade guides [5].

To overcome these limitations, instrumental methods such as spectrophotometers and colorimeters have been introduced to provide objective color measurements [4]. Among these, spectrophotometry is considered the reference standard because it quantifies tooth color using standardized Commission Internationale de l'Éclairage (CIE) L*a*b* coordinates and minimizes operator-dependent variability [6]. However, the high cost, limited accessibility, and additional clinical time associated with these devices restrict their routine use in dental practice.

Recent advances in artificial intelligence (AI), particularly deep learning and convolutional neural networks, have introduced promising alternatives for dental shade selection [7]. AI-based systems can analyze standardized digital photographs, perform automated color calibration, and predict the most appropriate tooth shade with minimal operator intervention. Such systems have the potential to improve consistency, reduce subjective errors, and facilitate rapid chairside shade determination [8]. Nevertheless, evidence comparing AI-integrated shade selection with conventional visual methods using spectrophotometric assessment as the reference standard remains limited. Therefore, the present study aimed to evaluate and compare the accuracy, agreement, and reliability of conventional visual and AI-integrated tooth shade selection methods against spectrophotometric shade matching. The primary objective was to compare the agreement and accuracy of both methods with the spectrophotometric reference. Secondary objectives were to assess the reliability of conventional visual shade selection, evaluate the diagnostic accuracy of both methods, compare shade-selection time, and determine the consistency of AI-generated shade predictions.

## Materials and methods

Study design and setting

This prospective, single-center, diagnostic method comparison study was conducted at the Department of Prosthodontics, K. M. Shah Dental College and Hospital, Sumandeep Vidyapeeth (Deemed to be University), Vadodara, India, between November 2021 and October 2022. A within-subject repeated-measures design was adopted, whereby each participant underwent all three shade assessments during a single clinical visit. The study adhered to the Standards for Reporting Diagnostic Accuracy Studies (STARD 2015) guidelines [9]. Ethical approval was obtained from the Institutional Ethics Committee (Reference no.: SVIEC/Dent/2021/Oct/115 dated October 04, 2021) before participant recruitment, and written informed consent was obtained from all participants.

Sample size and participant selection

Sample size estimation was performed using GPower version 3.1.9.7 (Heinrich-Heine-Universität Düsseldorf, Düsseldorf, Germany). Because the study generated both categorical shade classifications (VITA shade designations) and continuous color measurements (CIE L*, a*, and b* coordinates and derived ΔE00 values), the sample size calculation was based on the reliability of the continuous color measurements rather than on the categorical VITA shade labels. Assuming an expected intraclass correlation coefficient (ICC) of 0.85, a significance level of 0.05, and 90% statistical power, the required sample size was estimated to be 50 participants [10]. To account for an anticipated 10% loss or exclusion of participants, the final target sample size was increased to 55 participants. Categorical agreement between shade selections and the spectrophotometric reference was subsequently evaluated using Cohen's kappa and weighted kappa statistics.

Adults aged ≥18 years with at least one intact, unrestored, and fully erupted maxillary central incisor requiring shade evaluation were consecutively recruited throughout the study period. All participants meeting the eligibility criteria were invited to participate, and written informed consent was obtained before enrolment. No eligible participant declined participation. Teeth with restorations, intrinsic or extrinsic discoloration, developmental defects, recent bleaching, active caries, periodontal disease, or periapical pathologies were excluded. Participants who were unable to undergo standardized photography or spectrophotometric evaluation were also excluded.

Examiner calibration and clinical standardization

Two calibrated examiners (SC, RD) independently performed the conventional visual shade assessments. Color vision was verified using the Ishihara pseudo-isochromatic Color Vision Test (Kanehara Trading Inc., Tokyo, Japan) [11] and the Farnsworth-Munsell 100 Hue Test (X-Rite Incorporated, Grand Rapids, Michigan, USA) [12]. Examiner calibration was performed before commencement of the study on 15 non-study participants who were completely separate from the final study sample and were not included in the analysis. Calibration was continued until a weighted kappa coefficient of at least 0.80 was achieved. The calibration exercise was completed before participant recruitment and was not repeated during the study period. All shade assessments were performed in the same operatory under standardized daylight-corrected illumination (5500-6500 K; color rendering index >90), consistent with established recommendations for visual and instrumental tooth shade selection [3,6,13]. Participants removed brightly colored cosmetics and clothing accessories, and a neutral gray bib was used to minimize color reflection. Before shade assessment, the index tooth was cleaned using non-fluoridated pumice slurry and allowed to rehydrate for approximately five minutes.

Standardized photography

Standardized clinical photographs were obtained using the same Nikon digital single-lens reflex (DSLR) camera (Nikon Corp., Tokyo, Japan) equipped with a 105-mm macro lens and ring flash. Images were acquired in manual exposure mode using an aperture of f/22, a shutter speed of 1/160 s, and an ISO sensitivity of 100, with the flash operated at a fixed manual output throughout image acquisition. These parameters were selected based on established dental photography protocols recommending a relatively small aperture, low ISO sensitivity, and shutter speeds of approximately 1/125-1/200 s for standardized intraoral photography. The camera was positioned perpendicular to the labial surface of the maxillary central incisor at a fixed working distance of approximately 30 cm, with the tooth positioned centrally within the frame. White balance was fixed to the flash/daylight setting, and all images were captured in RAW format to preserve the original color information. A calibrated 18% neutral grey card and an X-Rite ColorChecker Classic (X-Rite Incorporated, Grand Rapids, Michigan, USA) were incorporated into each photograph to facilitate standardized color calibration during image processing. Images demonstrating poor focus, prominent glare or specular reflection, improper angulation, or motion artifacts were immediately repeated. This standardized acquisition protocol was used to maintain consistent illumination and color-calibration conditions across all participants; however, the use of a ring flash rather than a cross-polarized photographic system may have allowed some residual surface reflection and was therefore considered a methodological limitation.

Conventional visual shade evaluation

Conventional visual shade selection was performed independently by two calibrated examiners using the VITA Classical A1-D4 Shade Guide and VITA Toothguide 3D-MASTER (VITA Zahnfabrik H. Rauter GmbH & Co. KG, Bad Säckingen, Germany) under standardized daylight-corrected illumination, following established clinical protocols for visual shade matching [3,6]. Before assessment, the index tooth was cleaned using a non-fluoridated pumice slurry, rinsed with water, and maintained in a naturally hydrated state. The middle third of the labial surface of the maxillary central incisor was used as the reference area for shade matching. Shade tabs were presented in a randomized sequence to minimize examiner bias.

Each examiner compared the tooth with the shade tabs at eye level and a viewing distance of approximately 30-40 cm. Individual shade comparisons were limited to 5-7 seconds to reduce retinal fatigue and chromatic adaptation, with brief intervals of viewing a neutral grey background between successive comparisons when required, in accordance with established visual shade-matching protocols [3,6]. Examiners independently recorded the selected shade on standardized data collection forms without knowledge of each other’s observations or the AI and spectrophotometric findings. To assess intra-examiner reliability, the entire shade selection procedure was repeated after a minimum interval of 24 hours under identical clinical conditions. Inter-examiner reliability was determined by comparing the independent shade selections of both examiners.

AI-assisted shade evaluation

Standardized clinical photographs were analyzed using an AI-integrated deep learning system based on a convolutional neural network (CNN) for automated tooth shade classification. The AI workflow comprised four sequential stages: image preprocessing, color calibration, model inference, and automated shade prediction. Initially, the region of interest corresponding to the index tooth was automatically detected and cropped. The images then underwent standardized preprocessing, including white-balance correction, exposure normalization using the in-frame 18% neutral grey card, image resizing, and controlled reduction of specular reflections to minimize image-related variability.

Following preprocessing, color calibration was performed using the embedded color references. Device-dependent RGB values were corrected using the in-frame 18% neutral grey card and X-Rite ColorChecker Classic and subsequently transformed into the standardized Commission Internationale de l'Éclairage (CIE) L*a*b* color space [14]. This calibration procedure was intended to minimize variations arising from camera sensors and illumination conditions and to provide standardized color information for subsequent shade analysis.

The color-calibrated image was subsequently analyzed by the CNN-based deep learning model. The model generated a predicted tooth shade corresponding to the closest matching tab within the VITA Classical A1-D4 or VITA Toothguide 3D-MASTER shade system. In addition to the primary predicted shade, the model generated a confidence score and ranked alternative shade matches. Where supported by the AI platform, estimated CIE L*, a*, and b* coordinates in the CIE 1976 Lab* color space were also recorded for direct comparison with the spectrophotometric reference measurements [14]. All AI-generated outputs were recorded automatically. The investigator (PSP) remained blinded to the conventional visual shade selections and spectrophotometric findings during AI evaluation to minimize assessment bias.

Spectrophotometric reference standard

Objective shade determination was performed using the VITA Easyshade® V digital spectrophotometer (VITA Zahnfabrik H. Rauter GmbH & Co. KG, Bad Säckingen, Germany). The instrument was calibrated according to the manufacturer’s instructions before each clinical session. Three consecutive measurements were obtained from the middle third of the labial surface of the selected tooth, with probe repositioning between recordings, following the standardized clinical protocol recommended for this device [6]. The modal shade value and the mean CIE L*, a*, and b* coordinates were recorded as the reference standard. The operator (SM) performing the spectrophotometric measurements remained blinded to both the conventional visual and AI-generated results. For categorical agreement analysis, the spectrophotometer’s recorded shade designation was used as the reference category and directly matched to the corresponding VITA Classical or VITA 3D-MASTER shade category reported by the visual and AI methods.

Clinical sequence and control of tooth hydration

To minimize the potential influence of dehydration on tooth color, all shade assessments were performed using a standardized sequence during a single clinical visit. After cleaning with non-fluoridated pumice slurry, the index tooth was rinsed and allowed to rehydrate for approximately 5 minutes before commencement of shade assessment. The conventional visual assessment was then performed under standardized illumination by the two calibrated examiners, with the VITA Classical and VITA Toothguide 3D-MASTER shade systems assessed during the same hydrated period. The standardized clinical photograph was subsequently obtained, followed immediately by spectrophotometric measurement. The total assessment period was kept as short as practicable, and the tooth was not intentionally dried during any of the assessment procedures. To minimize progressive dehydration during the within-subject measurements, the sequence of the assessment methods was standardized, and the tooth was maintained in a naturally hydrated state throughout the procedure. The visual shade comparisons were limited to approximately 5-7 seconds per comparison, with brief intervals between successive observations, as previously described. The spectrophotometric assessment consisted of three consecutive measurements with probe repositioning between recordings. All procedures were completed during the same clinical visit, without prolonged mouth opening or unnecessary delays between assessments.

Outcome measures

The primary outcome was the agreement between conventional visual and AI-assisted shade selection methods and the spectrophotometric reference standard, evaluated using exact and weighted shade agreement and CIEDE2000 (ΔE00) color differences. The secondary outcomes included intra- and inter-examiner reliability, AI test-retest reliability, diagnostic accuracy, time required for shade selection, and AI confidence scores. Color differences were calculated using the CIEDE2000 (ΔE₀₀) formula [15], and clinically perceptible (ΔE₀₀ > 0.8) and acceptable (ΔE₀₀ > 1.8) thresholds were applied as previously established [13,16].

Data management and statistical analysis

Clinical data were recorded using standardized case record forms and entered independently into Microsoft Excel (Microsoft Corporation, Redmond, Washington, USA) using the double-entry method. Statistical analyses were performed using IBM SPSS Statistics (version 29, IBM Corp., Armonk, New York, USA). Data normality was assessed using the Shapiro-Wilk test. Continuous variables were presented as mean ± standard deviation or median (interquartile range), while categorical variables were expressed as frequencies and percentages. Agreement was evaluated using Cohen's kappa and weighted kappa. Pairwise comparisons were performed using the paired t-test or Wilcoxon signed-rank test as appropriate. Reliability was assessed using the intraclass correlation coefficient (ICC) and weighted kappa statistics. McNemar's test was used to evaluate the agreement between methods. Diagnostic accuracy was determined by calculating the sensitivity, specificity, positive predictive value, negative predictive value, and overall accuracy. Receiver operating characteristic (ROC) curve analysis was performed to calculate the area under the curve (AUC), and the optimal cut-off values were identified using the Youden index. Statistical significance was set at p < 0.05.

Bias control

To minimize bias, all participants underwent all three shade assessment methods under identical clinical conditions. Examiners performing conventional visual assessments and spectrophotometric measurements were blinded to each other's findings and the AI-generated results. The order of shade assessment methods was randomized, and standardized illumination, photography, calibration, and data entry procedures were maintained throughout the study.

## Results

In total, 55 participants were included in the final analysis. The mean age of the participants was 37.1 ± 11.3 years (range: 18-65 years). The study population comprised 30 (54.5%) males and 25 (45.5%) females. The right maxillary central incisor served as the index tooth in 30 (54.5%) participants, whereas the left maxillary central incisor was evaluated in 25 (45.5%) participants (Table 1).

**Table 1:** Baseline characteristics of the study participants (N = 55). Data are presented as mean ± standard deviation, range, or number (%), as appropriate, N = total number of participants, SD = standard deviation.

Characteristic		Value
Participants, n	Enrolled	55
Age, years	Mean ± SD	37.1 ± 11.3
	Range	18 – 65
Sex, n (%)	Female	25 (45.5%)
	Male	30 (54.5%)
Index tooth, n (%)	Right central incisor	30 (54.5%)
	Left central incisor	25 (45.5%)

The AI-integrated shade selection method demonstrated superior agreement with the spectrophotometric reference standard compared to the conventional visual method. The AI method achieved a Cohen's kappa value of 0.75 (95% CI: 0.63-0.86), indicating substantial agreement, and a weighted kappa of 0.99, indicating almost perfect agreement. Exact shade agreement was observed in 78.2% of the cases, with 99.0% agreement within one shade tab. In comparison, the conventional visual method showed moderate agreement with a Cohen's kappa of 0.43 (95% CI: 0.28-0.57) and a weighted kappa of 0.97, with exact shade agreement in 49.1% of cases and within-one-tab agreement in 92.7% of cases (Table 2).

**Table 2:** Agreement between conventional visual and AI-integrated shade selection methods and the spectrophotometric reference standard. Agreement was evaluated using unweighted Cohen's kappa and quadratic-weighted kappa with bootstrap 95% confidence intervals (3,000 resamples), Strength of agreement was interpreted as moderate (0.41–0.60), substantial (0.61–0.80), and almost perfect (0.81–1.00), CI = confidence interval, κ = kappa coefficient.

Method vs. spectrophotometric reference	κ (statistic)	95% CI	Strength of agreement
AI-integrated — unweighted Cohen's κ	0.75	0.63 – 0.86	78.1% (Substantial)
AI-integrated — weighted κ (quadratic)	0.99	0.98 – 0.99	99.0% (Almost perfect)
Conventional visual — unweighted Cohen's κ	0.43	0.28 – 0.57	49.1% (Moderate)
Conventional visual — weighted κ (quadratic)	0.97	0.92 – 0.98	92.7% (Almost perfect)

Reliability analysis demonstrated moderate to good reproducibility of the conventional visual method. Intra-examiner reliability showed a single-measure ICC of 0.71 (95% CI: 0.55-0.82) and an average-measure ICC of 0.83. Inter-examiner reliability demonstrated a single-measure ICC of 0.74 (95% CI: 0.60-0.84) and an average-measure ICC of 0.85. The AI-integrated method exhibited excellent test-retest reliability, with a single-measure ICC of 0.97 (95% CI: 0.95-0.98) and an average-measure ICC of 0.98 (Table 3).

**Table 3:** Intra-examiner, inter-examiner, and AI test-retest reliability. Reliability was assessed using the intraclass correlation coefficient based on a two-way random-effects model with absolute agreement, *p < 0.05 were considered statistically significant, ICC = intraclass correlation coefficient, CI = confidence interval, df = degree of freedom.

Reliability domain	ICC (single)	95% CI	F test (df)	p-value	ICC (average-k)
Intra-examiner (visual, test–retest ≥24 h)	0.71	0.55 – 0.82	F(54,54) = 5.97	< 0.001*	0.83
Inter-examiner (visual, Examiner 1 vs 2)	0.74	0.60 – 0.84	F(54,54) = 6.77	< 0.001*	0.85
AI test–retest (same calibrated image)	0.97	0.95 – 0.98	F(54,54) = 62.94	< 0.001*	0.98

The AI-integrated method demonstrated lower color differences relative to the spectrophotometric reference than the conventional visual method. The mean ΔE00 value for the AI method was 1.42 ± 1.25, whereas the conventional visual method showed a mean ΔE00 of 1.76 ± 1.44. Clinically unacceptable color differences (ΔE00 >1.8) were observed in 23.6% of the AI-assisted assessments compared to 45.5% of the conventional visual assessments (Table 4).

**Table 4:** Color difference (ΔE00) between each shade selection method and the spectrophotometric reference standard. Continuous variables are presented as mean ± standard deviation and median (interquartile range), Percentages indicate the proportion of observations exceeding the perceptibility (ΔE00 > 0.8) and acceptability (ΔE00 > 1.8) thresholds, ΔE00 = CIEDE2000 color difference, IQR = interquartile range, SD = standard deviation.

Method	Mean ± SD	Median (IQR)	% ΔE00 > 0.8	% ΔE00 > 1.8
AI-integrated	1.42 ± 1.25	1.18 (0.66–1.73)	69.1%	23.6%
Conventional visual	1.76 ± 1.44	1.56 (0.59–2.40)	67.3%	45.5%
Spectrophotometer (self-reference error)	1.39 ± 1.27	1.10 (0.63–1.73)	65.5%	25.5%

Paired comparisons demonstrated that the AI-integrated method produced significantly lower ΔE00 values than the conventional visual method (mean difference = −0.34, 95% CI: −0.57 to −0.11; p = 0.020). No statistically significant difference was observed between the AI method and the spectrophotometric reference (p = 0.446), whereas the conventional visual method differed significantly from the reference standard (mean difference = 0.37, 95% CI: 0.13-0.60; p = 0.006) (Table 5).

**Table 5:** Paired comparison of color differences between shade selection methods. Pairwise comparisons were performed using the Wilcoxon signed-rank test, Values of *p < 0.05 were considered statistically significant, CI = confidence interval, W = Wilcoxon signed-rank statistic, ΔE00 = CIEDE2000 color difference.

Paired comparison	Mean difference (95% CI)	Test statistic (W)	p-value
AI vs. Conventional visual ΔE00	-0.34 (−0.57 to −0.11)	472	0.020*
AI vs. Spectrophotometer ΔE00	0.03 (−0.05 to 0.11)	679	0.446*
Conventional visual vs. Spectrophotometer ΔE00	0.37 (0.13 to 0.60)	442	0.006

For the detection of clinically perceptible color mismatch (ΔE00 >0.8), the AI-integrated method achieved a sensitivity of 94.3%, specificity of 75.0%, positive predictive value of 86.8%, negative predictive value of 88.2%, and an overall diagnostic accuracy of 87.3%. The conventional visual method demonstrated a lower diagnostic performance, with a sensitivity of 82.9%, specificity of 60.0%, and overall accuracy of 74.5%. Similar findings were observed for the clinically acceptable threshold (ΔE00 >1.8), where the AI method maintained an overall diagnostic accuracy of 87.3% compared with 72.7% for the conventional visual method (Table 6).

**Table 6:** Diagnostic accuracy of conventional visual and AI-integrated shade selection methods using spectrophotometric assessment as the reference standard. Diagnostic performance was evaluated using sensitivity, specificity, positive predictive value (PPV), negative predictive value (NPV), and overall accuracy with 95% confidence intervals, Spectrophotometric measurements served as the reference standard, CI = confidence interval, ΔE00 = CIEDE2000 colour difference.

Spectrophotometric reference	Methods	Sensitivity % (95% CI)	Specificity % (95% CI)	PPV % (95% CI)	NPV % (95% CI)	Accuracy % (95% CI)
ΔE00 > 0.8	AI-integrated	94.3 (81.4 – 98.4)	75.0 (53.1 – 88.8)	86.8 (72.7 – 94.2)	88.2 (65.7 – 96.7)	87.3 (76.0 – 93.7)
	Conventional visual	82.9 (67.3 – 91.9)	60.0 (38.7 – 78.1)	78.4 (62.8 – 88.6)	66.7 (43.7 – 83.7)	74.5 (61.7 – 84.2)
ΔE00 > 1.8	AI-integrated	71.4 (45.4 – 88.3)	92.7 (80.6 – 97.5)	76.9 (49.7 – 91.8)	90.5 (77.9 – 96.2)	87.3 (76.0 – 93.7)
	Conventional visual	85.7 (60.1 – 96.0)	68.3 (53.0 – 80.4)	48.0 (30.0 – 66.5)	93.3 (78.7 – 98.2)	72.7 (59.8 – 82.7)

McNemar's test demonstrated no significant disagreement between the AI-integrated method and the spectrophotometric reference at either the perceptibility threshold (χ² = 0.57, p = 0.450) or acceptability threshold (χ² = 0.00, p = 1.000). In contrast, the conventional visual method demonstrated significant disagreement at the acceptability threshold (χ² = 6.67, p = 0.010), indicating a comparatively lower agreement with the reference standard (Table 7).

**Table 7:** McNemar test for paired disagreement between shade selection methods and the spectrophotometric reference standard. Paired categorical comparisons were performed using McNemar's test, Effect size is expressed as the odds ratio with 95% confidence intervals, Values of *p < 0.05 were considered statistically significant, OR = odds ratio, CI = confidence interval, χ² = chi-square statistic, ΔE00 = CIEDE2000 color difference.

Level	Methods	McNemar χ² (test vs. reference)	p-value	Odds ratio, (95% CI)
ΔE00 > 0.8	AI-integrated	0.57	0.450	49.50 (8.60 – 284.78)
	Conventional visual	0.07	0.789	7.25 (2.07 – 25.41)
ΔE00 > 1.8	AI-integrated	0.00	1.000	31.67 (6.07 – 165.07)
	Conventional visual	6.67	0.010*	12.92 (2.52 – 66.30)

Receiver operating characteristic curve analysis demonstrated the excellent diagnostic performance of the AI-integrated method for identifying clinically unacceptable color mismatch, with an AUC of 0.932 (95% CI: 0.856-1.000). The optimal cut-off value determined using the Youden index was 1.63, corresponding to a sensitivity of 85.7% and a specificity of 87.8%. The conventional visual method also demonstrated good diagnostic performance, with an AUC of 0.895 (95% CI: 0.794-0.997) and an optimal cut-off value of 2.49, although its overall discriminative ability was lower than that of the AI-integrated method (Table 8; Figure 1).

**Table 8:** Receiver operating characteristic curve analysis of colour difference values for identifying clinically unacceptable shade mismatch. Receiver operating characteristic curve analysis was performed using continuous ΔE00 values, The optimal cut-off value was determined using the Youden index. Sensitivity and specificity are reported at the optimal cut-off, AUC = area under the receiver operating characteristic curve, CI = confidence interval, ΔE00 = CIEDE2000 colour difference.

Method (continuous ΔE00 score)	AUC	95% CI	Optimal cut-off (Youden)	Sensitivity % / Specificity % at cut-off
AI-integrated	0.932	0.856 – 1.000	1.63	85.7 / 87.8
Conventional visual	0.895	0.794 – 0.997	2.49	78.6 / 95.1

**Figure 1:**
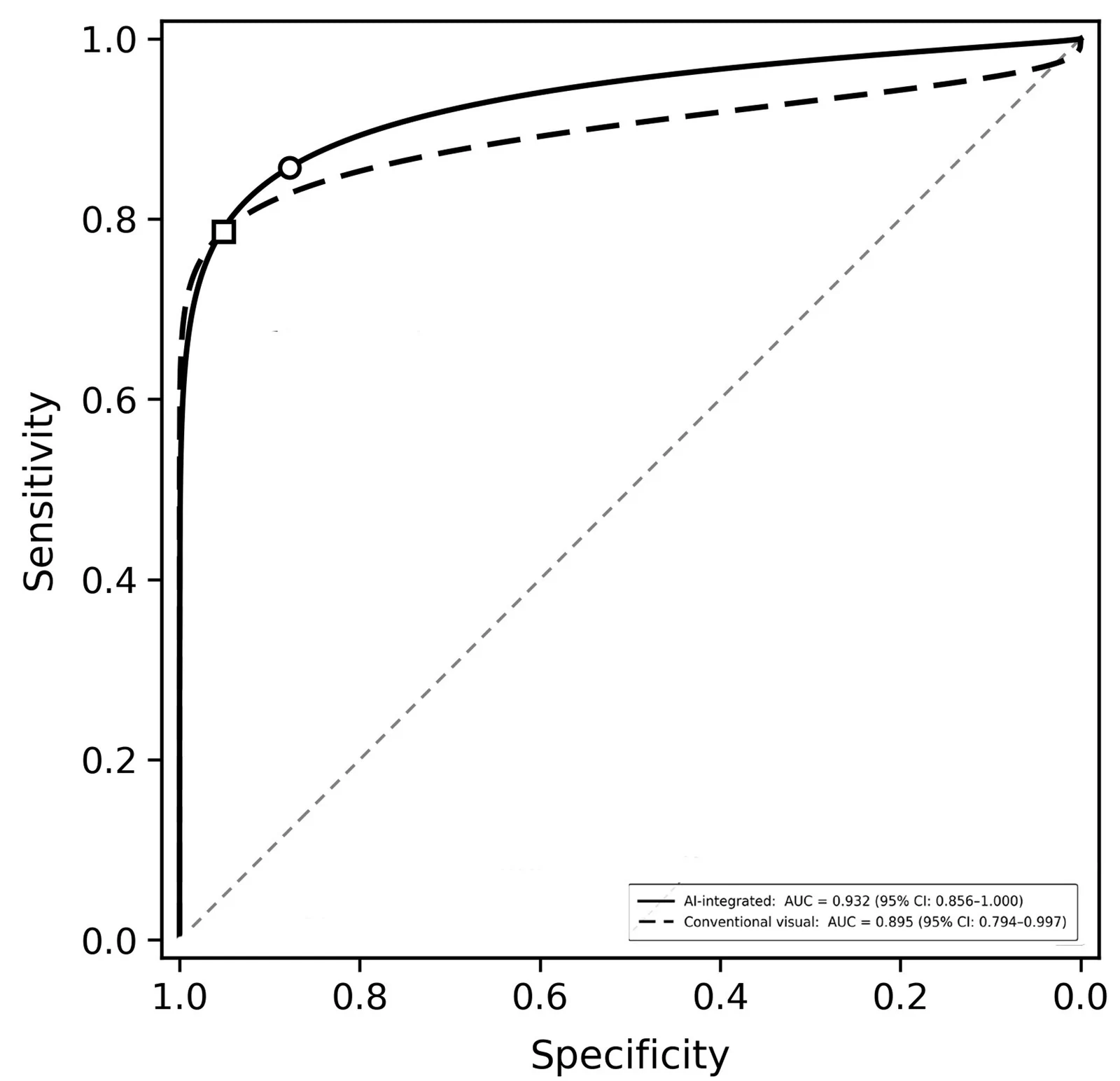
Receiver operating characteristic (ROC) curves for discrimination using continuous ΔE00 scores. The AI-integrated method demonstrated a higher area under the curve (AUC = 0.932; 95% CI: 0.856–1.000) than the conventional visual method (AUC = 0.895; 95% CI: 0.794–0.997). AI = artificial intelligence, AUC = area under the receiver operating characteristic curve.

## Discussion

Accurate tooth-shade selection remains one of the most critical determinants of esthetic success in restorative and prosthodontic dentistry [1]. This study compared conventional visual shade selection with an AI-integrated method using spectrophotometric measurements as the reference standard. The findings demonstrated that the AI-integrated approach consistently outperformed the conventional visual assessment in terms of agreement, color accuracy, reliability, and diagnostic performance.

The AI-integrated method demonstrated a substantially higher agreement with the spectrophotometric reference standard than the conventional visual method. Furthermore, the weighted kappa value of 0.99 indicated almost perfect agreement between the AI-generated shade predictions and the spectrophotometric measurements, whereas the conventional method achieved a slightly lower weighted agreement. These findings suggest that AI can effectively reduce the subjective variability associated with human color perception by analyzing standardized digital images using objective computational algorithms [7]. The high within-one-tab agreement observed for the AI method further indicates that AI-assisted shade selection can produce clinically acceptable shade recommendations with excellent consistency.

The difference between unweighted and weighted agreement highlights the importance of considering the ordinal relationship between dental shade categories. The very high weighted agreement observed for both assessment approaches likely reflects the fact that most discrepancies occurred between adjacent or closely related shade tabs. Weighted kappa accounts for the degree of disagreement and therefore gives greater consideration to near-agreements than unweighted kappa. Consequently, the high weighted agreement should not be interpreted as indicating equivalent exact shade selection. Rather, the findings suggest that although exact categorical matching was less consistent, particularly with conventional visual assessment, the discrepant selections were generally close to the reference shade. This distinction is clinically relevant because a near-adjacent shade discrepancy represents a smaller color deviation than a mismatch involving widely separated shade categories.

Li et al. [17] developed a genetic algorithm-optimized backpropagation neural network for computerized dental color matching, demonstrating improved prediction precision and robustness compared with conventional neural network models. Subsequently, Chen et al. [18] introduced a machine-learning-based shade-matching system incorporating the hue, saturation, and value (HSV) color model with fuzzy decision technology, achieving an overall shade-matching accuracy of 99.78%. These advances highlight the ability of AI to improve the consistency and accuracy of tooth shade determination and provide a foundation for the development of contemporary deep-learning-based shade selection systems.

Reliability analysis also favored the AI-integrated method. While conventional visual assessment demonstrated moderate to good intra- and inter-examiner reliability, the AI system showed excellent test-retest reliability with an ICC of 0.97, reflecting highly reproducible shade predictions from repeated analyses of standardized images. This finding is expected because AI algorithms are unaffected by physiological factors that influence human observers, including retinal fatigue, color adaptation, visual acuity, and examiner experience [7]. Once image acquisition and color calibration have been standardized, AI systems produce consistent outputs with minimal operator-dependent variability.

Wei et al. [19] evaluated a novel computer color-matching system based on an improved back-propagation neural network and demonstrated significantly greater color reproduction accuracy than the conventional visual method. The AI-based system produced a significantly lower mean color difference (ΔE = 1.89 ± 0.75) compared with visual shade selection (ΔE = 3.54 ± 1.11; p < 0.01), suggesting that neural network-based algorithms can improve the precision and consistency of tooth shade matching.

The AI-assisted method also demonstrated a significantly lower color difference (ΔE00) than the conventional visual method. Importantly, no significant difference was observed between the AI predictions and the spectrophotometric reference standard, whereas the conventional visual assessment showed significantly greater color discrepancies. These findings indicate that AI-generated shade predictions closely approximate objective spectrophotometric measurements and may therefore improve the esthetic predictability of restorative procedures [20]. Lower ΔE00 values are clinically relevant because they reduce the likelihood of visible color mismatch, restoration remakes, additional chairside adjustments, and patient dissatisfaction [13].

Image processing and machine learning techniques have also been successfully applied for automated tooth shade recognition. Justiawan et al. [21] compared different machine learning algorithms for tooth color classification using HSV and CIE L*a*b* color moment features and reported that the k-nearest neighbors (KNN) algorithm achieved the highest classification accuracy of 97.5% with rapid computation time. These findings further support the potential of AI-based image analysis for objective and efficient tooth shade determination.

The superior diagnostic performance of the AI-integrated method further supports its clinical utility. AI demonstrated higher sensitivity, specificity, positive predictive value, negative predictive value, and overall diagnostic accuracy than conventional visual assessments for both perceptibility and acceptability thresholds. Similarly, the ROC analysis showed an excellent AUC of 0.932 for AI compared with 0.895 for the conventional method, indicating a greater discriminative ability for identifying clinically unacceptable shade mismatches. Collectively, these findings suggest that AI-assisted shade selection may serve as a reliable adjunct for clinicians, particularly for esthetically demanding restorative procedures.

A literature review by Tabatabaian et al. [22] reported that instrumental methods, particularly spectrophotometers, provide greater accuracy and repeatability than conventional visual shade selection by minimizing the subjective errors associated with human color perception. This review also highlighted the superior performance of the VITA Easyshade system as one of the most reliable digital shade-matching devices. Similarly, a systematic review and meta-analysis by Hardan et al. [20] demonstrated that computerized shade selection methods produce significantly lower color differences (ΔE) and fewer incorrect shade matches than conventional visual shade selection, particularly when digital photography is used for shade matching. These findings support the present results, in which the AI-assisted image analysis yielded significantly lower ΔE00 values and superior agreement with the spectrophotometric reference standard.

The improved performance of the AI observed in the present study may be attributed to several methodological factors. First, standardized photography using fixed camera settings and color calibration minimizes image variability before AI analysis. Second, conversion of color information into the CIE L*a*b* color space allowed objective color quantification independent of camera characteristics. Finally, deep learning algorithms simultaneously analyze multiple color and texture features that are difficult for the human visual system to consistently evaluate, thereby reducing observer-dependent variability and improving reproducibility.

From a clinical perspective, AI-assisted shade selection has the potential to improve the communication between clinicians and dental laboratories by providing objective and reproducible shade information. The integration of AI into routine restorative workflows may reduce remakes, shorten chairside adjustment time, improve esthetic outcomes, and enhance patient satisfaction. As AI analysis requires only standardized digital photographs, it may represent a practical and cost-effective adjunct for dental practices that do not have routine access to spectrophotometers. However, AI should currently be regarded as an adjunct rather than a replacement for clinical judgment, particularly in complex esthetic cases, where factors such as translucency, fluorescence, and surface texture continue to require clinician assessment.

The present study had certain limitations. This study was conducted at a single center with a relatively modest sample size, which may limit the generalizability of our findings. Only the maxillary anterior teeth were evaluated under standardized clinical conditions, and the performance of the AI system under varying lighting conditions, camera systems, or diverse populations was not investigated. Furthermore, this study evaluated a single AI workflow without comparing it to multiple commercially available AI platforms. Future multicenter studies involving larger and more diverse populations, different restorative materials, and external validation of various AI systems are required to confirm these findings and to establish standardized clinical protocols for AI-assisted tooth shade selection.

## Conclusions

The AI-integrated tooth shade selection method demonstrated higher agreement, lower colour differences, greater reproducibility, and improved diagnostic performance compared with conventional visual assessment when evaluated against spectrophotometric measurements. These findings suggest that AI-assisted shade selection may provide a useful adjunct to conventional clinical shade assessment. However, interpretation should remain cautious given the modest sample size, single-centre design, and absence of external validation. Further multicentre studies involving larger and more diverse populations and independent validation are warranted to confirm the reproducibility and generalizability of these findings.
